# A Double Threat: Concurrent Non-ST-Elevation Myocardial Infarction and Transient Ischemic Attack Symptoms Revealing Triple-Vessel Coronary Artery Disease

**DOI:** 10.7759/cureus.88628

**Published:** 2025-07-23

**Authors:** Roger Lin, Milan Regmi

**Affiliations:** 1 Internal Medicine, Southeast Health Medical Center, Dothan, USA

**Keywords:** left ventricular thrombus, methamphetamine use, mural thrombus, myocardial infarction, transient ischemic attack, triple vessel disease, virchow triad

## Abstract

Impaired ventricular wall motion and reduced ejection fraction after an acute myocardial infarction promote blood stasis - especially in hypokinetic or akinetic segments - activating coagulation pathways, platelet aggregation, and thrombus formation. Concurrent endothelial injury from ischemia further exposes thrombogenic surfaces, and if the thrombus embolizes, it can trigger serious neurologic events such as stroke or transient ischemic attack (TIA). We describe the case of a 56-year-old man with hypertension and methamphetamine use who presented with chest pain, dyspnea, and transient left-sided weakness. Work-up revealed elevated troponin, severely calcified triple-vessel coronary disease, and an apical mural thrombus with an ejection fraction of 45%, confirmed on transthoracic echocardiography, with subsequent paroxysmal atrial flutter. Methamphetamine use, known to accelerate atherosclerosis and heighten thromboembolic risk, likely compounded his coronary disease. The patient was anticoagulated with enoxaparin, bridging to warfarin, and discharged for follow-up imaging and surgical evaluation. This case highlights the need for heightened vigilance, prompt anticoagulation, and thorough cardiac evaluation for mural thrombus and embolic complications in methamphetamine-associated myocardial injury, even when initial cerebrovascular imaging is negative.

## Introduction

Symptomatic mural thrombi can lead to devastating complications such as stroke. The pathophysiology of mural thrombus formation is governed by Virchow’s triad: (i) blood stasis - seen when infarcted ventricular segments become hypokinetic or akinetic, (ii) endothelial injury - from subendocardial necrosis that exposes thrombogenic collagen and tissue factor, and (iii) hypercoagulability - augmented by post-infarction inflammation that activates platelets and the clotting cascade [1,2]. Methamphetamine use adds a synergistic insult: recurrent catecholamine surges precipitate coronary vasospasm, accelerate atherosclerosis, and promote endothelial dysfunction, creating a milieu in which intraventricular thrombi and systemic emboli are increasingly reported [3]. Consequently, left ventricular (LV) thrombus remains a well-recognized complication of myocardial infarction and may culminate in embolic stroke or transient ischemic attack with long-term disability.

We present an interesting case of triple-vessel coronary artery disease complicated by an apical mural thrombus and transient ischemic attack in a patient with recent methamphetamine use, underscoring the additive pro-thrombotic risk conferred by stimulant-associated vascular injury.

## Case presentation

A 56-year-old man with gastro-oesophageal reflux disease, hiatal hernia, essential hypertension, polysubstance use disorder, and a remote transient ischaemic attack (age 23 years) presented to the emergency department with a 3-day history of intermittent, sharp left-central chest pain radiating to the left arm, accompanied by exertional dyspnoea, dizziness, and transient left-sided weakness. The symptoms began after he smoked crystal methamphetamine 3 days prior to presentation while drinking at a bar; he reported four episodes of methamphetamine use in the preceding 18 months, daily beer consumption, and a one-pack-per-day cigarette habit. He denied cocaine or other illicit drug use and took no prescribed medications, though he ingested an unspecified antihypertensive tablet just before activating emergency medical services.

On arrival, he was afebrile with blood pressure 144/83 mmHg, heart rate 77 beats min⁻¹, respiratory rate 20 breaths min⁻¹, and oxygen saturation 97% on ambient air. Laboratory studies showed plasma glucose 121 mg dL⁻¹, aspartate aminotransferase 41 U L⁻¹, high-sensitivity troponin-I 2158 ng L⁻¹ (reference <20 ng L⁻¹), and B-type natriuretic peptide 231 pg mL⁻¹; the complete blood count, basic metabolic panel, lipid profile (triglycerides 163 mg dL⁻¹), and urinalysis were otherwise unremarkable. Urine toxicology was positive for amphetamines. A chest radiograph was normal, and a 12-lead electrocardiogram demonstrated normal sinus rhythm without ST-segment deviation. Serial high sensitivity troponin trended up to 2436 ng L⁻¹ (reference <20 ng L⁻¹) in 4 hours (Table 1).

**Table 1:** Troponin high sensitivity level during hospitalization

Time	Troponin High Sensitivity (ng L^-1^)	Reference Range
On admission	2,158	<20 ng L^-1^
2 hrs follow-up	2,186	<20 ng L^-1^
4 hrs follow-up	2,436	<20 ng L^-1^

The patient had been admitted for chest pain the previous year, but scheduled outpatient stress testing and echocardiography were never completed. He lives independently and was fully functional at baseline, without supplemental oxygen, but described marked limitation in activities of daily living since symptom onset.

Transient weakness was evaluated with immediate imaging studies, including CT head and CT angiogram, which ruled out large ischemic or hemorrhagic stroke. By the time of ED presentation, the patient's weakness was already resolved.

**Figure 1:**
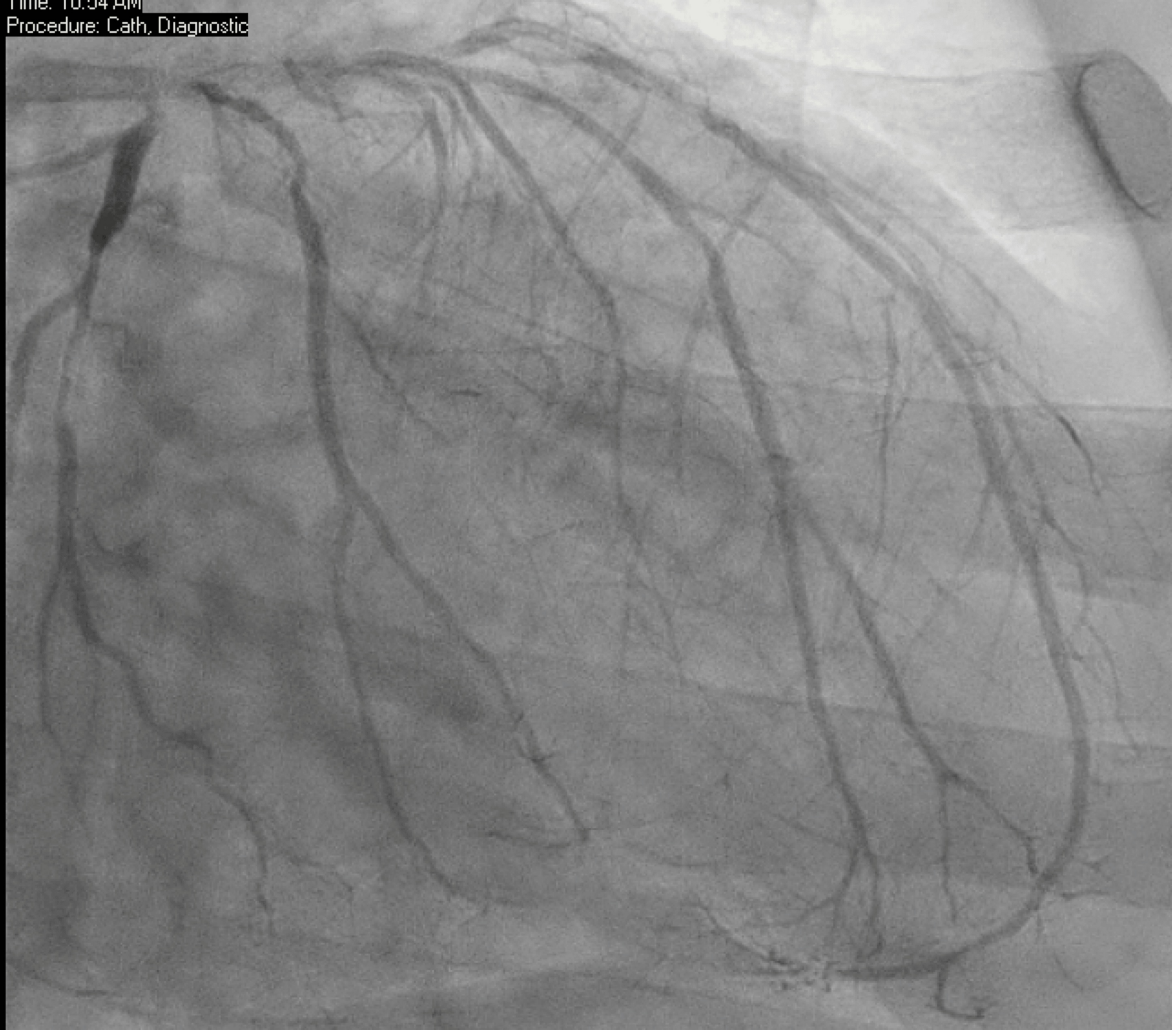
Heavily calcified coronary artery disease.

**Figure 2:**
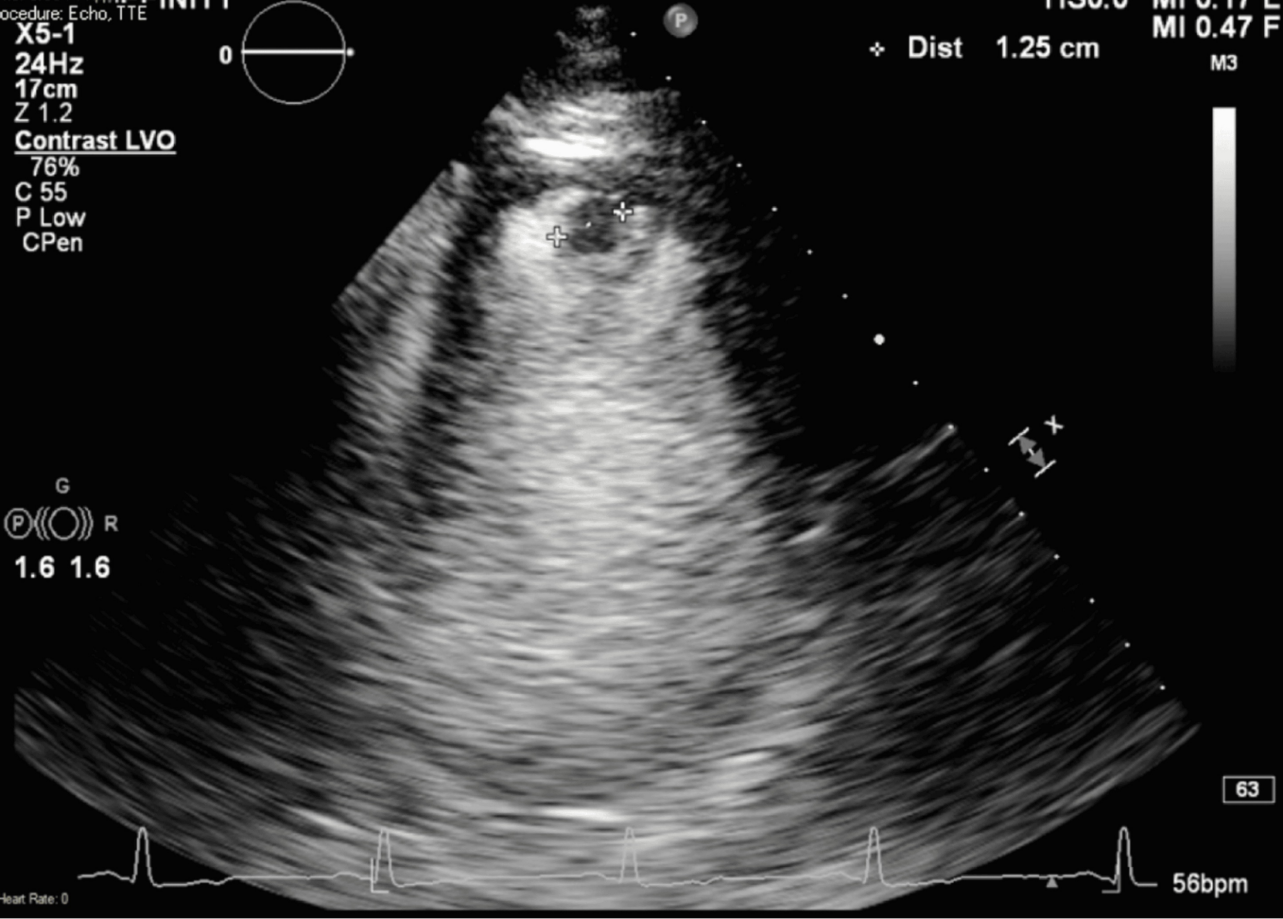
Transthoracic echocardiography showing evidence of mural apical filling defect consistent with left ventricular apical thrombus.

Due to concern of non-ST elevation myocardial infarction (NSTEMI) type 1, cardiac catheterization was done urgently to reveal apical mural thrombus with severely calcified three-vessel coronary artery disease with ejection fraction (EF) of 45% (Figure 1). During the cardiac catheterization, the patient developed paroxysmal atrial flutter. Apical mural thrombus was confirmed with transthoracic echocardiography (TTE) (Figure 2), which showed apical filling defect with apical wall motion abnormality, suggesting left anterior descending artery (LAD)distribution with apical mural thrombus.

At discharge, the patient was prescribed guideline-directed medical therapy (GDMT) for heart failure, including lisinopril for afterload reduction and carvedilol for beta-blockade. Ranolazine was continued for angina symptom control. For coronary artery disease, the patient was maintained on atorvastatin for lipid management and aspirin for antiplatelet therapy. He was started on warfarin with enoxaparin sodium bridging due to the apical mural thrombus. His financial constraints precluded the use of a direct oral anticoagulant (DOAC). He was discharged with warfarin and was scheduled to have a follow-up TTE to reassess the size and shape of the apical mural thrombus to further stratify his risk of proceeding with coronary bypass surgery.

The patient was adherent with warfarin, and repeat TTE reported no thrombus seen in the left ventricular (LV) apex with perflutren lipid microsphere enhancement. He was cleared by Cardiology and underwent elective four-vessel coronary-artery bypass grafting (CABG×4). The procedure was uncomplicated, and he was extubated the same day, but on postoperative day (POD) 2, he developed acute left facial droop, dysarthria, and hemiparesis. Head CT perfusion demonstrated a large right middle-cerebral-artery territory infarct, and angiography revealed chronic right internal-carotid-artery occlusion with extensive collateralization and a small anterior-cranial-fossa dural arteriovenous fistula. Neurologic deficits improved rapidly with supportive care and intensive physiotherapy; by POD 5, he had near-complete motor recovery, chest tubes and pacing wires were removed without incident, and diuresis resolved postoperative fluid overload. On POD 6, he ambulated independently without supplemental oxygen and performed activities of daily living unassisted. He was discharged to an inpatient rehabilitation facility on warfarin 7.5 mg nightly (target international normalized ratio (INR)2-3) with therapeutic enoxaparin bridging and plans for serial imaging and outpatient cardiopulmonary rehabilitation.

## Discussion

Methamphetamine is increasingly recognized as a potent cardiotoxic and neurotoxic agent. Population-based and mechanistic studies show that even intermittent exposure markedly increases the risk of both acute myocardial infarction and ischemic stroke, largely through catecholamine-mediated vasospasm, accelerated atherosclerosis, hypertension, and a pro-thrombotic milieu [4]. In our patient, the temporal relationship between crystal-methamphetamine inhalation and symptom onset, coupled with his limited prior use, strongly suggests that methamphetamine acted as the inciting factor superimposed on traditional risks (hypertension, smoking, alcohol).

Methamphetamine’s vasculotoxic effects likely accelerated existing three-vessel coronary disease and precipitated the non-ST-elevation myocardial infarction (NSTEMI). The resulting apical hypokinesis and moderately reduced ejection fraction created the classic substrate for left ventricular (LV) mural thrombus formation - blood stasis within an akinetic apex - and therefore fulfilled two elements of Virchow’s triad (stasis ± endothelial injury) [2]. Concomitant catecholamine surges, platelet activation, and endothelial dysfunction from methamphetamine further amplify thrombotic risk [4].

The patient’s transient facial droop and hemiparesis before surgery, along with his large right-middle-cerebral-artery infarct after coronary artery bypass grafting (CABG), highlight the embolic potential of LV thrombi. Small, platelet-rich emboli may pass undetected on conventional CT imaging yet produce brief neurological deficits; advanced techniques such as continuous transcranial Doppler can identify such microembolic signals, underscoring why clinical suspicion must remain high even when initial imaging is unrevealing [5]. 

Current expert consensus recommends an oral anticoagulant for at least 3 months for LV thrombus after myocardial infarction, with repeat imaging to document resolution before stopping therapy [6]. Warfarin remains acceptable when direct oral anticoagulants (DOACs) are economically prohibitive, provided low-molecular-weight heparin bridging is used until a therapeutic INR is achieved, as was done in this case [6]. 

Despite successful thrombus resolution and guideline-directed timing of surgery, the patient experienced a peri-operative stroke - a complication reported in 2-5% of CABG procedures. Established risk factors include prior transient ischemic attack, carotid or aortic arch disease, atrial arrhythmias, and prolonged cardiopulmonary-bypass time [7]. In retrospect, his chronic right-internal-carotid-artery occlusion and peri-procedural atrial flutter likely compounded his baseline risk. 

Importantly, the burden of methamphetamine-associated cardiovascular disease - mirroring findings in other substance-impacted populations - is disproportionately borne by underserved communities. For instance, the NYC4H cohort (mean age 61.6 years, 62.9% male) exhibited high rates of polysubstance use (31.1%), mental health issues (20.7%), and lack of family support (14.5%), with 57.6% experiencing at least one social adversity. Cardiovascular risk assessment should therefore increasingly incorporate these social determinants to guide tailored prevention and management strategies [8].

## Conclusions

This case underscores the complex interplay between substance abuse, particularly methamphetamine use, and cardiovascular pathology. The presence of triple-vessel coronary artery disease, left ventricular mural thrombus, and transient ischemic symptoms in our patient highlights the potential for methamphetamine to accelerate atherosclerosis and promote thrombus formation through multiple mechanisms. Prompt recognition of mural thrombi in the context of myocardial infarction is crucial, as early anticoagulation can mitigate the risk of embolic complications. Clinicians should maintain a high index of suspicion for cardiac sources of embolism in patients presenting with neurologic symptoms and a history of substance use, especially when traditional risk factors for thromboembolic events are present. Early diagnosis, aggressive risk factor modification, and close follow-up are essential to improving outcomes in such patients.
